# TIV Vaccination Modulates Host Responses to Influenza Virus Infection that Correlate with Protection against Bacterial Superinfection

**DOI:** 10.3390/vaccines7030113

**Published:** 2019-09-12

**Authors:** Angela Choi, Ioanna Christopoulou, Xavier Saelens, Adolfo García-Sastre, Michael Schotsaert

**Affiliations:** 1 Department of Microbiology, Icahn School of Medicine at Mount Sinai, New York, NY 10029, USA; Angela.Choi@Icahn.mssm.edu; 2 Graduate School of Biomedical Sciences, Icahn School of Medicine at Mount Sinai, New York, NY 10029, USA; 3 Global Health and Emerging Pathogens Institute, Icahn School of Medicine at Mount Sinai, New York, NY 10029, USA; 4 VIB-UGent Center for Medical Biotechnology, Department of Biomedical Molecular Biology, Ghent University 9000-9052 Ghent, Belgium; ioannachristopoulou@googlemail.com (I.C.); Xavier.Saelens@ugent.vib.be (X.S.); 5 Department of Medicine, Division of Infectious Diseases, Icahn School of Medicine at Mount Sinai, New York, NY 10029, USA; 6 The Tish Cancer Institute, Icahn School of Medicine at Mount Sinai, New York, NY 10029, USA

**Keywords:** influenza, TIV, bacterial superinfection, *Staphylococcus aureus*, macrophage, eosinophil, neutrophil

## Abstract

Background: Influenza virus infection predisposes to secondary bacterial pneumonia. Currently licensed influenza vaccines aim at the induction of neutralizing antibodies and are less effective if the induction of neutralizing antibodies is low and/or the influenza virus changes its antigenic surface. We investigated the effect of suboptimal vaccination on the outcome of post-influenza bacterial superinfection. Methods: We established a mouse vaccination model that allows control of disease severity after influenza virus infection despite inefficient induction of virus-neutralizing antibody titers by vaccination. We investigated the effect of vaccination on virus-induced host immune responses and on the outcome of superinfection with *Staphylococcus aureus*. Results: Vaccination with trivalent inactivated virus vaccine (TIV) reduced morbidity after influenza A virus infection but did not prevent virus replication completely. Despite the poor induction of influenza-specific antibodies, TIV protected from mortality after bacterial superinfection. Vaccination limited loss of alveolar macrophages and reduced levels of infiltrating pulmonary monocytes after influenza virus infection. Interestingly, TIV vaccination resulted in enhanced levels of eosinophils after influenza virus infection and recruitment of neutrophils in both lungs and mediastinal lymph nodes after bacterial superinfection. Conclusion: These observations highlight the importance of disease modulation by influenza vaccination, even when suboptimal, and suggest that influenza vaccination is still beneficial to protect during bacterial superinfection in the absence of complete virus neutralization.

## 1. Introduction

Over the course of a lifetime, humans build up immunity to influenza viruses through natural infection and vaccination. Currently licensed vaccines aim at the induction of virus neutralizing antibodies. The gold correlate of protection so far is an in vitro surrogate assay for virus neutralization based on the ability of serum antibodies to prevent virus from agglutinating red blood cells, which is called hemagglutination inhibition (HI). The presence of such antibodies is associated with a reduction in the likelihood of morbidity upon exposure to influenza virus [1]. Protection provided by neutralizing antibodies is, however, limited in time, since influenza virus can change its antigenic surface through antigenic drift and shift, or because levels of neutralizing antibodies wane over time. There is an abundance of literature that describes mechanisms other than virus-neutralizing antibodies that can contribute to protection against influenza-associated morbidity and mortality [2,3,4,5,6]. In fact, it is very likely that, in the absence of virus-neutralizing antibodies, influenza virus-induced disease is modulated instead of prevented in the human host by non-neutralizing pre-existing immunity. As a result, host immune responses to the virus are also skewed during infection by pre-existing immunity. This is often not reflected in preclinical animal models for influenza virus infection since mostly naïve animals are used. To better reflect the human situation, we developed a mouse model in which we induce influenza-specific pre-existing immunity that allows virus replication but confers disease protection. Infection-permissive pre-existing immunity is induced by intramuscular vaccination with trivalent inactivated virus vaccine (TIV) adjuvanted with alhydrogel (alum). With this model, we study the initial host immune response to infection with a sublethal dose of H1N1 influenza virus that is homologous to the H1N1 component of the TIV. We show that TIV is disease protective, even in the absence of detectable virus-neutralizing antibodies, and skews host immune responses upon infection. It has been described that influenza virus infection can predispose the host for bacterial pneumonia [7,8,9], and bacterial superinfection during influenza outbreaks is a major cause of influenza-related death [10]. Therefore, we also investigated the effect of pre-existing immunity provided by TIV on the outcome of *Staphylococcus aureus* superinfection. Modulation of influenza-related disease by TIV was able to prevent mortality during superinfection, and this was associated with enhanced neutrophil recruitment to the lungs and mediastinal lymph nodes. Therefore, TIV vaccination was not only able to protect from disease after initial influenza virus infection, but also indirectly during secondary bacterial superinfection. The results described in this manuscript provide insights as to how influenza-specific pre-existing immunity can ameliorate the host response to both influenza virus infection and bacterial superinfection in the absence of detectable neutralizing antibodies.

## 2. Materials and Methods

### 2.1. Study Design

**Figure 1:**
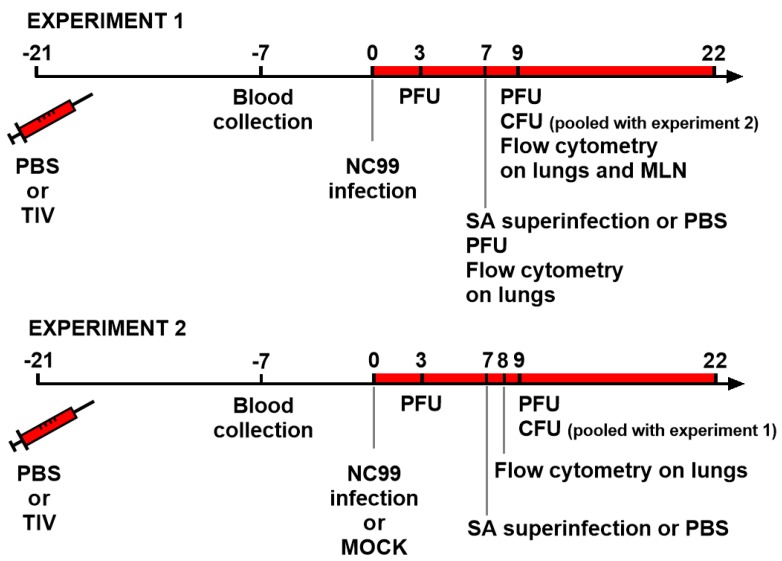
Outlines for Experiment 1 and 2. Mice were TIV vaccinated or control vaccinated with PBS 21 days before infection with NC99 H1N1 virus. In Experiment 2, a mock challenged control group was also included. Blood for serology was collected 14 days after vaccination. Lungs were collected for virus titration at 3 dpi, 7 dpi (only Experiment 1) and 9 dpi. Bacterial superinfection was given at 7 dpi and bacterial loads were determined at 9 dpi. Flow cytometry for investigating host immune responses was done on samples collected at 7 dpi (Experiment 1), 8 dpi (Experiment 2) and 9 dpi (Experiment 1). PBS: phosphate buffered saline, TIV: trivalent inactivated virus vaccine, SA: *Staphylococcus aureus*, PFU: virus plaque forming units, CFU: bacterial colony forming units.

For this study, two experiments were carried out. The schematic outline for Experiment 1 and Experiment 2 are shown in Figure 1. Briefly, for Experiment 1, fifty mice were either vaccinated with TIV (*n* = 25) or administered with PBS (*n* = 25) on day 21. Both groups were then challenged with a sublethal dose of NC99 H1N1 virus three weeks later (day 0). Body weights were monitored for up to 14 days post infection. One week after virus infection (day 7), remaining mice in the two groups were further divided in half to infect with *S. aureus* (SA) or a control treatment with PBS. Experiment 2 was set up in the same way as Experiment 1, but with addition of control mock infection with allantoic fluid, which brought the total number of mice to 96. For serology, blood was collected 14 days post vaccination (day 7) in both experiments. For lung viral titers, lungs were harvested at 3, 7 and 9 days post infection (dpi) for Experiment 1 and at 3 and 9 dpi for Experiment 2. In both experiments, bacterial loads were measured from lungs harvested at 2 days post *S. aureus* infection (9 days post H1N1 infection). To investigate host immune responses, flow cytometry was performed on samples collected on different time points. In Experiment 1, lung samples were collected at 7 and 9 days post H1N1 infection (day 7 and day 9). Additionally, at day 9, mediastinal lymph nodes were collected. Flow cytometry analysis in Experiment 2 was performed only on lungs that were harvested at 8 days post H1N1 infection (day 8).

### 2.2. Mice, Vaccination and Serology

Female Balb/c mice aged 6–8 weeks were obtained from Charles River Laboratories (Balb/c AnNCrl) and housed under specified pathogen-free conditions with food and water *ad libitum*.

Trivalent inactivated virus vaccine (TIV) was the human Aventis-Pasteur vaccine (2005–2006 formula) containing the antigens from the following influenza viruses: A/New Caledonia/20/99 H1N1 virus, A/New York/55/2004 and B/Jiangsu/10/2003. Vaccine was obtained from BEI resource and mixed with alhydrogel (Alum, Invivogen, San Diego, CA, USA) before incubation at 4 °C according to the manufacturer’s directions. Vaccine was injected once via the intramuscular route with a BD 300 μL insulin syringe in the quadriceps muscles of the hind legs (100 μL divided over both legs). The administered vaccine dose corresponds to 3 μg of each hemagglutinin type in the vaccine per mouse. Control groups were injected with phosphate buffered saline (PBS) mixed with Alum.

Blood was collected at the indicated time points via submandibular bleeding and serum was prepared by allowing the blood to clot at room temperature. Anti-HA antibody responses were measured by enhanced luminescent immunosorbent assay (ELISA) and hemagglutination inhibition (HI) assay. For quantification of HA-specific total IgG levels by ELISA, 96 well NUNC Maxisorp plates were coated with baculovirus-derived recombinant HA from A/New Caledonia/20/99 in bicarbonate buffer at 4 °C overnight. After washing and blocking with 4% milk for 1 h at room temperature, serum samples diluted 1/100 in PBS with 0.05% Tween20 are allowed to bind ELISA antigen for 1.5 h at room temperature. Plates were washed three times with PBS (0.05% Tween20) and incubated with sheep-derived anti-mouse IgG serum conjugated to horse-radish peroxidase (GE Healthcare, Amersham, UK). After a final washing step, tetramethylbenzidine substrate (Sigma-Aldrich, San Diego, CA, USA) was used to estimate levels of HA-specific mouse IgG by measuring the OD450 with the OD650 as a reference.

Hemagglutination inhibition was performed as previously described [5]. Briefly, four volumes of receptor destroying enzyme (RDE, Vibrio cholera filtrate, Sigma Aldrich, San Diego, CA, USA) were added to each volume of mouse serum. After overnight incubation at 37 °C, sera were heat-inactivated at 56 °C for 30 min in citrate buffer. Four hemagglutination units of A/New Caledonia/20/99 were mixed with twofold dilutions of treated sera in a final volume of 50 μL. Mixtures of virus and diluted serum were allowed to bind for 1h at room temperature before 50 μL of 0.5% chicken red blood cell suspension was added. HI titers were read after 1h incubation on ice.

### 2.3. Experimental Infection with Influenza Virus and Staphylococcus aureus

Three weeks after vaccination, mice were challenged intranasally with 50 μL of a sublethal dose of egg-grown A/New Caledonia/20/99 H1N1 virus (400 plaque forming units, PFU) preparation diluted in PBS under mild ketamine/xylazine anesthesia. Mock-challenged mice received diluted allantoic fluid instead of virus. For bacterial superinfections, *Staphylococcus aureus* was grown in Bacto tryptic soy broth (BD, Sparks, MD, USA) to an OD600 of 1.4. This guaranteed that bacterial growth was in the log phase. 10^8^ colony forming units (CFU) were administered intranasally at 7 days post influenza virus infection in 50 μL under mild ketamine/xylazine anesthesia. Control mice received the same volume of PBS instead of bacteria suspension. Body weight loss was measured on a daily base as a read-out for morbidity. All experiments were approved by and performed according to the guidelines of the Icahn School of Medicine at Mount Sinai Institutional Animal Care and Use Committee (IACUC-2017-0330). The methods used were carried out in accordance with the approved guidelines. Mice were euthanized when they had reached the ethical endpoint of 25% body weight loss.

### 2.4. Lung Virus Titers

Lung virus titers were quantified by plaque assays as described before [11]. Briefly, the mice were sacrificed, and the lungs were removed aseptically at the indicated time points and homogenized in PBS with a Benchmark Beadblaster 24 (Benchmark Scientific, Edison, NJ, USA). Lung homogenates were cleared by centrifugation (16,000× *g*) at 4 °C and stored at −80 °C. For plaque assays, 250 μL of tenfold dilutions in PBS of lung homogenate suspensions were incubated on confluent monolayers of Madin Darbin Canine Kidney cells at 37 °C. After 1 h incubation, the inoculum was removed by aspiration and cells were overlaid with 2% oxoid agar (Oxoid, Basingstoke, UK) mixed with an equal volume of NaHCO3-buffered 2xMEM supplemented with DEAE/Dextran and TPCK-treated trypsin (1 μg/mL). Cells were incubated for 48 h at 37 °C and 5% CO2. Plaque formation was visualized by staining of cell surfaces after fixation with 4% formaldehyde (5 min at room temperature). For staining, cells were incubated with a 1/1000 dilution of post challenge mouse serum followed by incubation with 1/1000 diluted sheep anti-mouse serum conjugated to horse radish peroxidase (GE Healthcare) and addition of TrueBlue substrate (KPL—Seracare, Milford, MA, USA).

### 2.5. Lung Bacterial Titers

Lungs were collected aseptically at the indicated time points and forced over a sterile 40 μm filter (Sigma, St. Louis, MO, USA) with the back of a 1 mL syringe. Lung bacterial titers were quantified by homogenizing lungs in PBS and plating different dilutions on tryptic soy agar bacterial plates. After overnight incubation at 37 °C, colony forming units (CFU) were counted.

### 2.6. Flow Cytometry

#### 2.6.1. Sample Preparation

Lungs and peribronchial lymph nodes were collected at the indicated time points. Lungs were cut into cubes of one cubic mm and digested with Type IV Collagenase (Sigma, St. Louis, MO, USA) in RPMI (Gibco, Grand Island, NY, USA) for 30 min at 37 °C. After incubation, single cell suspensions were made by forcing lung tissue over a 40 μm filter (Sigma) with the back of a 1 mL syringe. In a similar way, lymph nodes were pooled and forced over a 40 μm filter (Sigma) with the back of a 1 mL syringe to obtain single cell suspensions. Cells were stained in 100 μL volume of FACS buffer (PBS + 0.5% bovine serum albumin (BSA) and 2 mM EDTA (Sigma)) with a viability dye (eFluor 520 and eFluor 780, eBiosciences, San Diego, CA, USA) and antibodies (all from BD Pharmingen unless mentioned otherwise) that target the following surface markers as mentioned in the text: CD11b (clone M1/70, APC), CD11c (clone HL3, PE-Cy7), SiglecF (clone E50-2440, PE-CF594), MerTK (clone DS5MMER, PE-Cy7 from eBioscience, CD64 (clone X54-5/7.1, PE), MHCII (clone M5/114.15.2, eFluor 450 from eBioscience), Ly6G (clone 1A8-Ly6g, eFluor 450 from eBioscience). Staining was done at room temperature for 30 min in the dark. Cells were washed twice with FACS buffer before analysis on a Beckman Coulter Gallios Flow cytometer. For estimation of absolute cell numbers, counting beads (Thermofisher, Eugene, OR, USA) were added to the samples.

#### 2.6.2. Analysis of Flow Cytometry Data

Flow cytometry data analysis was performed using FlowJo version X.0.7 (Treestar Inc., Ashland, OR, USA) for classical hierarchical gating using two-dimensional plots. For unsupervised analysis of samples collected at 8 days post infection in Experiment 2, compensated flow cytometry files were loaded in FlowJo for preprocessing: Single live cells were gated and exported as new FCS 3.0 files. Exported flow cytometry data were then further analyzed using the R/Bioconductor packages flowCore and matrixStats. Marker intensities were arcsinh-transformed (cofactor = 150) and scaled between values 0 to 1 for visualization. Packages ConsensusClusterPlus and FlowSOM are used for cell clustering and visualized using the packages Rtsne, ggplot2, pheatmap and RcolorBrewer.

### 2.7. Statistical Analysis

Statistical analyses were performed with Graphpad Prism version 7.00 for Windows (GraphPad Software, San Diego, CA, USA) and with the R language and environment for statistical computing, R Development Core Team, 2009 (R Foundation for Statistical Computing, Vienna, Austria (ISBN 3-900051-07-0, URL http://www.R-project.org). The statistical tests used for computing significance levels are mentioned in the text. Significance levels are mentioned in the text or indicated with single asterisk (*p* < 0.05) or double asterisks (*p* < 0.01) in figures when not mentioned in the text.

## 3. Results

### 3.1. Single TIV Vaccination Does Not Result in Efficient Induction of Virus-Neutralizing HI Antibodies and Is Infection-Permissive

**Figure 2:**
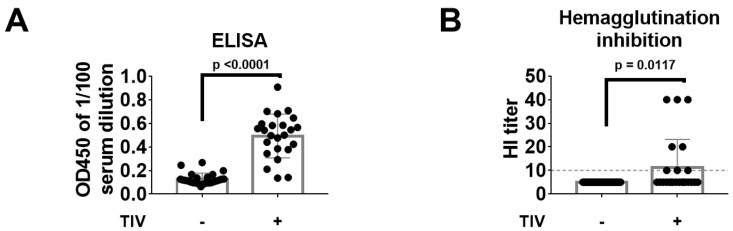
Serology at 14 days post vaccination. (**A**) Serum was 1/100 diluted and probed for NC99 H1 HA-specific antibodies by ELISA using recombinant NC99 H1 HA as a coating antigen. (**B**) Hemagglutination inhibition titers were determined in RDE-treated serum samples. Grey dotted line represents the detection limit. Bars represent means and error bars represent standard deviation. Differences between groups were tested using unpaired *t*-tests.

Blood was collected two weeks after vaccination by submandibular bleeding. Single administration of TIV with the equivalent of 3 mg hemagglutinin (HA) was able to induce NC99 H1-specific HA ELISA titers in most samples tested (Figure 2A). When these serum samples were then tested for their capacity to inhibit virus-mediated hemagglutination of red blood cells, a proxy assay for virus neutralization, only 33% of the samples from TIV-vaccinated mice tested had an HI titer at or above the detection limit (HI titer = 10) (Figure 2B).

**Figure 3:**
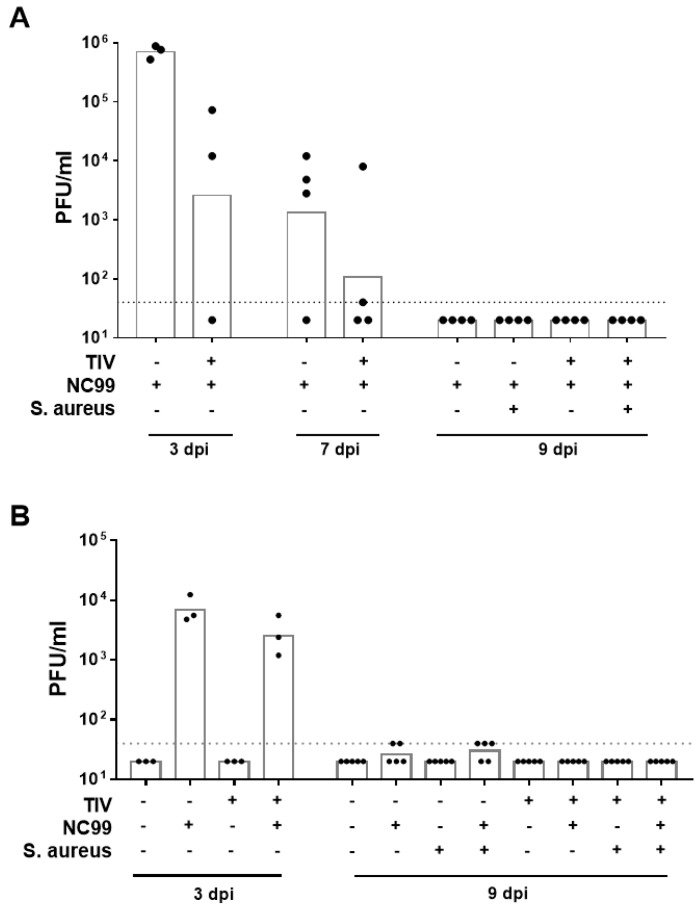
Lung viral titers at different days post infection with NC99 H1N1 virus for (**A**) Experiment 1 and (**B**) Experiment 2. Dotted line represents the limit of detection. Bars represent geometric means.

The poor induction of detectable neutralizing antibodies by TIV vaccination was further reflected by the presence of detectable lung virus titers in vaccinated mice at 3 days post infection (dpi) in both experiments (Figure 3A,B). Vaccination, however, reduced lung virus titers about tenfold (Experiment 2) up to 100-fold (Experiment 1) compared to control vaccinated animals at 3 dpi and virus was controlled in three out of four mice by 7 dpi (Figure 3A,B). No lung virus titers above detection limit were observed by 9 dpi (Figure 3A,B). In conclusion, TIV vaccination did not result in sterilizing immunity but aided virus clearance from the lungs of infected mice.

### 3.2. TIV Vaccination Results in Protection from Mortality and Morbidity after H1N1 Infection and Prevents Mortality upon Bacterial Superinfection

**Figure 4:**
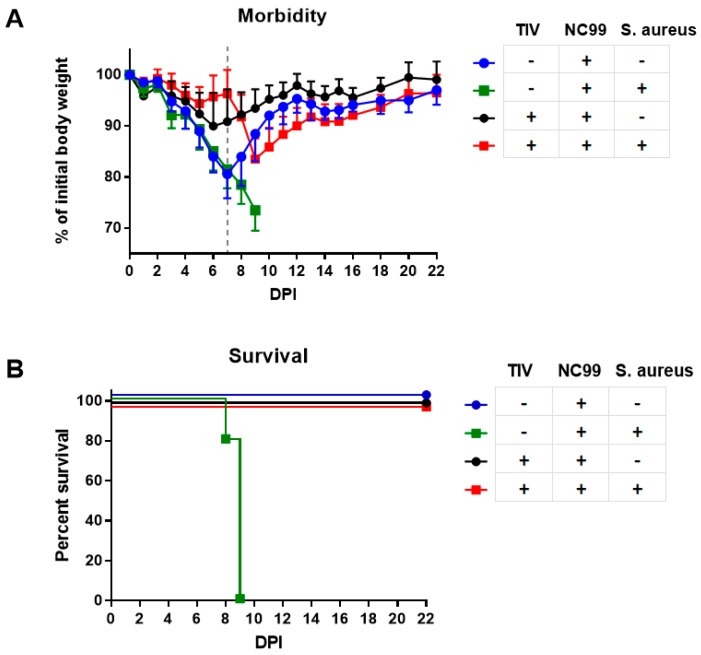
(**A**) Morbidity and (**B**) mortality after infection with NC99 H1N1 virus, with or without *S. aureus* superinfection for Experiment 1. Dotted line in (**A**) represents 7 dpi, when *S. aureus* was given to mice. Error bars represent standard deviation.

**Figure 5:**
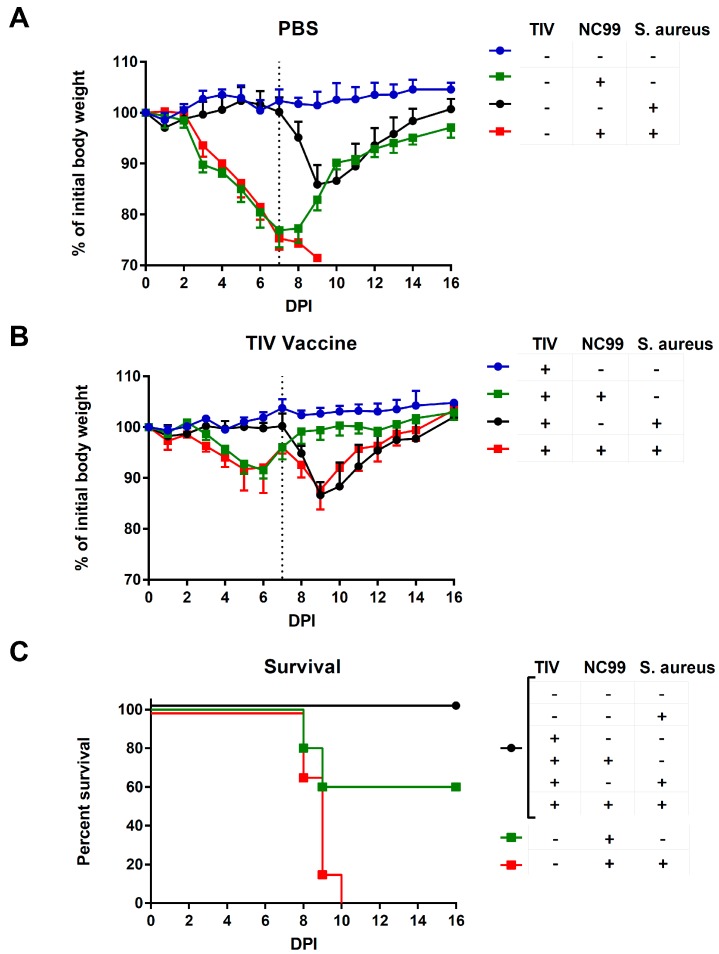
(**A**) Morbidity in control vaccinated or (**B**) TIV vaccinated animals and (**C**) mortality after infection with NC99 H1N1 virus with and without superinfection with *S. aureus* for Experiment 2. Dotted line in (**A**) and (**B**) represents 7 dpi, when *S. aureus* was given to mice. Error bars represent standard deviation.

**Figure 6:**
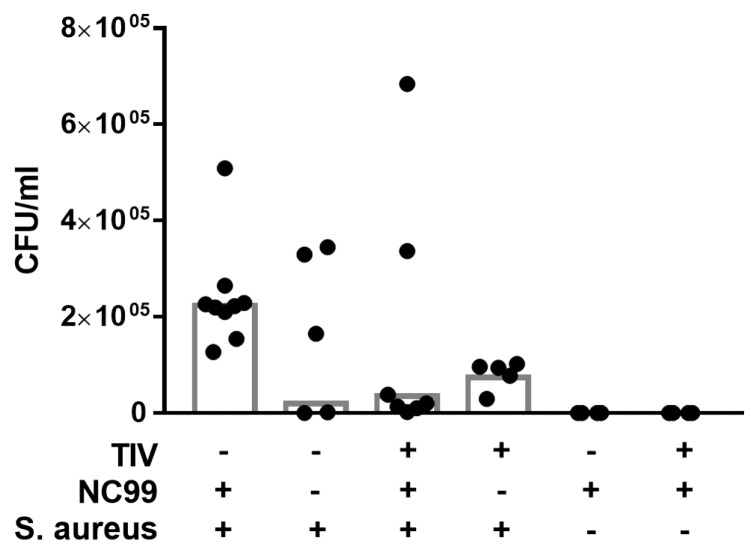
Bacterial titers measured at 9 dpi with NC99 H1N1 virus (2 days post bacterial superinfection). Bars represent geometric means. Data are pooled for Experiment 1 and 2.

Control-vaccinated mice lost 20% to 25% on average of their initial body weight after infection with NC99 virus (Figure 4A and Figure 5A). The challenge was sublethal for all mice in Experiment 1 but was lethal to some mice in Experiment 2 (Figure 4B and Figure 5C). Virus-induced morbidity was further enhanced and resulted in mortality when mice were given a superinfection with 10^8^ colony forming units of *S. aureus* at 7dpi (Figure 4A,B and Figure 5A,C). Bacterial infection without previous influenza infection resulted in 15% of body weight loss and mice started to recover after 2 days (Figure 4A and Figure 5A). Vaccination induced control of morbidity after NC99 H1N1 influenza virus infection and prevented mortality during bacterial superinfection (Figure 4A,B and Figure 5B,C). There seems to be a trend of better control of bacterial load in vaccinated mice compared to control vaccinated mice 2 days after bacterial superinfection (Figure 6); however, there was too much variation in bacterial titers to draw solid conclusions on this point with the number of mice we sampled. A similar observation was made by Chaussee et al. in a mouse model for post-influenza superinfection with *Streptococcus pyogenes* and vaccination [8].

### 3.3. TIV Vaccination Modulates the Host Immune Response to Both Influenza Virus Infection and S. aureus Superinfection

**Figure 7:**
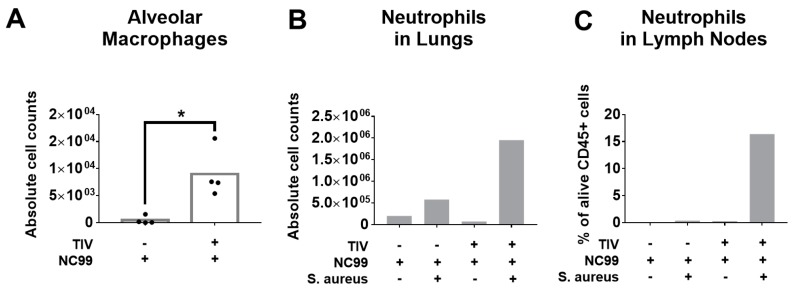
TIV vaccination affects levels of alveolar macrophages and neutrophils after NC99 H1N1 infection and bacterial superinfection (Experiment 1). (**A**) Absolute numbers of alveolar macrophages were quantified at 7 dpi with NC99 H1N1 virus in single cell suspensions of whole lung tissue Bars represent means. (**B**) Absolute cell counts of neutrophils in single cell suspensions of pooled lung tissue from 3 mice/group and (**C**) relative levels of neutrophils in single cell suspensions of pooled lung-draining lymph nodes from 3 mice/group measured at 9 dpi with influenza virus (2 days post bacterial superinfection). * *p* < 0.05.

The infection-permissive nature of TIV vaccination suggests that the host mounts a host immune response to the infection. It has been described for the Balb/c mouse model that alveolar macrophages are depleted after influenza virus infection [12,13,14]. This can predispose to severe disease during bacterial superinfection. In Experiment 1, we therefore quantified the absolute number of alveolar macrophages (alive CD45+ CD11c+ SiglecF+ CD11b- CD64+ MerTK+) in lung tissue at 7 dpi with influenza virus. We observed that despite virus replication, alveolar macrophages are protected to some extent during influenza virus infection in TIV vaccinated mice, but not in control vaccinated mice (Figure 7A). Neutrophils are important for host defense during bacterial infections and their function can be impaired by influenza infection [15]. Two days later, at 9 dpi with influenza virus, which is 2 days after bacterial superinfection, we monitored neutrophil levels in both lung tissue and lung-draining lymph nodes. Bacterial superinfection with *S. aureus* resulted in a modest increase of neutrophils in control vaccinated animals, which was almost fourfold higher in vaccinated animals (Figure 7B). The enhanced levels of neutrophils (alive Ly6G+ CD11b+) in lungs of TIV vaccinated animals are also reflected in the lung-draining lymph nodes (Figure 7C).

**Figure 8:**
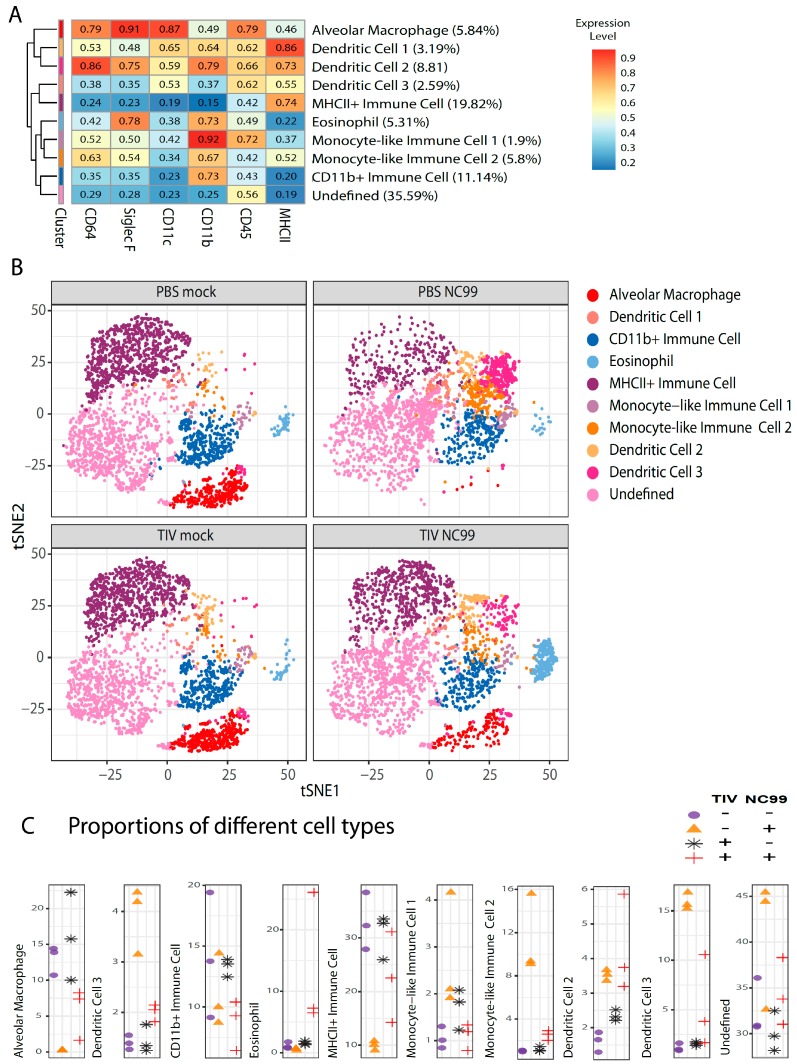
Unsupervised analysis of immune cell populations in the lung at 8 days post influenza virus infection for Experiment 2. Single cell suspensions from mouse lungs (*n* = 3/group) were stained for viability and surface markers. (**A**) Live cells were used to define immune cell populations by clustering cells based on their mean fluorescence intensities for different surface markers that are mentioned at the bottom of the heat map. Every row represents the surface expression profile for one cluster. Rows were reshuffled so that hierarchical tree distance reflects cluster similarity. Z-scored mean fluorescence intensities are given for the respective clusters in the heatmap. Relative abundance of different clusters is given between parentheses after the cluster names. (**B**) t-SNE plots for the four experimental groups. Every plot represents subsamples of 3000 cells/mouse with *n* = 3 mice/group. (**C**) Proportions of the different immune cell populations. Every dot represents an individual mouse and different symbols are used for the different experimental conditions.

In Experiment 2, we further characterized the effect of TIV vaccination on virus-host immune responses at 8 dpi with influenza virus (no samples from animals that received bacterial superinfection were included). We decided to do an unsupervised approach to cluster cells with similar expression levels of surface markers (CD45, CD11b, CD11c, CD64, SiglecF and MHCII) using the FlowSOM algorithm [16] after “classical” hierarchical gating on single live CD45+ cells. This makes it possible to visualize (the abundance of) multiple cell types present in different samples in one plot. Another advantage is that no prior knowledge of the cellular composition of the samples is required. Although already used for selecting immune cells before starting the unsupervised analysis, expression levels of CD45 were also considered for clustering since expression levels can differ based on the type of immune cell. With this antibody panel, we defined 10 clusters (Figure 8A). Using this approach, we could clearly separate alveolar macrophages from other immune cells (Figure 8B) and observed that alveolar macrophages are depleted at 8 days post infection in control vaccinated animals but not in TIV vaccinated animals (Figure 8B,C). This confirmed our observations for 7 dpi in Experiment 1. The advantage of using dimension reduction implementations like t-SNE is that different cell populations can be visualized simultaneously without prior knowledge of the cell populations present in the sample (Figure 8B). This allows a more complete overview of the dynamics of different immune cell populations at a certain time point for the different conditions we tested. As such, we were able to define a cell population that was enriched uniquely in TIV-vaccinated animals and that was almost absent in control vaccinated animals after influenza virus infection. This cell population was also not enriched in TIV or control vaccinated animals without influenza virus infection. The surface marker expression profile identified this population as eosinophils (CD11b+ SiglecF+ CD11c-). On the other hand, CD11b- DCs, characterized as CD11b- CD11c+ CD64-SiglecF- population with intermediate MHCII levels, seemed to be enriched in control vaccinated animals after influenza virus infection. The myeloid compartment in the influenza virus-infected lung is a complex mixture of immune cells from different lineages and ontogeny [17,18]. Nevertheless, the limited number of markers used in this analysis allows to illustrate the dynamics of other immune populations other than eosinophils and alveolar macrophages after influenza virus infection and how they are skewed by TIV vaccination. We named cells that expressed CD11b in combination with another monocyte-associated markers “monocyte-like immune cells” (Figure 8). Some of these monocyte-like immune cells have intermediate levels of CD11c and other markers typically associated with DCs and/or alveolar macrophages like MHCII, CD11b, SiglecF or CD64 (Figure 8A). This suggests that some of the monocyte-like immune cell populations observed in Figure 8B represent lung-infiltrating monocytes that transition into DCs or alveolar macrophages. Compared to mock infected animals, proportions of the different “monocyte-like immune cell” populations changed after infection in TIV vaccinated animals, which was more pronounced in control vaccinated animals after infection, reflecting the higher morbidity that the latter group experienced after infection (Figure 8B,C).

## 4. Discussion

Split vaccines are widely used to try to prevent influenza virus-related morbidity and mortality in the human population. Induction of virus-neutralizing antibodies against circulating virus strains correlates with protection. However, even if vaccine strains and circulating viruses match antigenically, levels of neutralizing antibodies in vaccinees can be too low to be measured by in vitro assays like the hemagglutination inhibition assay or microneutralization assays. The reasons for low antibody titers can be multiple, ranging from poor induction of initial antibody responses to waning antibody levels over time, even after successful vaccination. In the absence of sufficient neutralizing antibodies, virus replication cannot be prevented, and the host can still induce immune responses to the infection. In this work, we showed that protection from disease provided by influenza vaccination with a human TIV does not necessarily correlate with induction of neutralizing antibodies in sera as measured by inhibition of hemagglutination of red blood cells. Therefore, assays other than hemagglutination inhibition are needed to fully assess the protective effect of TIV vaccination, as well as to understand the contribution of pre-existing immunity. In the absence of detectable hemagglutination inhibitory (HI) antibody titers in sera, TIV-mediated protection was infection-permissive, meaning that virus replication was allowed to some extent. We also showed that pre-existing immunity provided by TIV vaccination in the absence of detectable HI titers in sera skews host responses to virus infection. In Balb/c mice, experimental infection with influenza virus results in death of alveolar macrophages [12,13,14]. TIV vaccination prevented complete loss of alveolar macrophages, despite the presence of replicating virus. This may suggest that in TIV vaccinated mice virus replication is more restricted, allowing macrophages to survive where virus is not replicating or replicating at lower titers. 

Severe influenza pathogenesis can be the result of pneumonia caused by superinfection with bacterial pathogens like *Streptococcus pneumoniae* and *Staphylococcus aureus* [10]. In fact, re-analysis of efficacy studies of bacterial vaccines used during the 1918 influenza pandemic showed that vaccine-induced antibacterial immunity correlated with protection from pneumonia and death [19]. Preventing pathogenesis during influenza infection by vaccination also protects from bacterial superinfection [8,20,21] and antibacterial vaccination can protect during post-influenza bacterial superinfection [22]. However, influenza infection can also make antibacterial vaccination less effective in the context of bacterial superinfection [23]. From preclinical animal models it is known that predisposition of influenza virus-infected hosts to bacterial superinfection is linked to alveolar macrophage death as well as to alveolar macrophages and other phagocytes becoming unresponsive to innate stimuli by exposure to interferon induced by the prior virus infection [7,12,20,24]. Contrary to PBS vaccinated animals, virus replication in TIV vaccinated mice did not result in detectable levels of type I interferons and reduces induction of antiviral IFNγ-producing T cell responses (Choi et al., manuscript in preparation), and therefore alveolar macrophages may not be exposed to interferon to the same extent. In our model, *Staphylococcus aureus* infection alone was not lethal, whereas superinfection after prior influenza infection was lethal in mice that received PBS, but not in TIV-vaccinated animals. In line with the enhanced morbidity observed after influenza virus infection in PBS-vaccinated mice compared to TIV mice, we observed more infiltration of monocyte-like immune cells in the lungs of PBS mice at 7 dpi. It has recently been described that a high ratio of lung-infiltrating monocytes over tissue-resident alveolar macrophages correlates with high pathogenicity in mice during influenza virus infection [25] and expression of CCR2, a marker associated with monocyte lineages, correlates with increased susceptibility to post-H1N1 *Staphylococcus aureus* superinfection by affecting dendritic cell functionality [26].

Interestingly, TIV vaccination resulted in enhanced infiltration of neutrophils in influenza virus infected lungs. Higher levels of neutrophils contribute to protection during bacterial pneumonia by phagocytosis and induction of anti-bacterial responses [27]. Neutrophil recruitment during post-H1N1 bacterial superinfection with *S. pneumoniae* and *S. aureus* relies on the production of IL17 and neutrophil attractants CXCL1 and CXCL2, and both are negatively affected by virus-induced type I interferon [28,29,30,31]. Reduction of virus replication and IFN production by vaccination can explain why TIV mice have more neutrophil influx upon bacterial superinfection. The importance of neutrophil infiltration in mediastinal lymph nodes for protection is unclear but they may shape humoral immune responses through interaction with B cells [32]. 

The recruitment of eosinophils into the lungs after respiratory infection of immunized hosts is dogmatically associated with negative outcome of disease, e.g., during infections with respiratory syncytial virus [33]. However, eosinophils have the molecular arsenal to fight bacterial infections [34] and have recently been shown to correlate with protection during influenza virus infections by both direct antiviral effects [35] and antigen-presentation to T cells [36]. Ongoing research in our laboratory is looking into a potential role for alveolar macrophages in recruiting eosinophils and neutrophils during infection in alum-adjuvanted, TIV-vaccinated mice, for example through the secretion of chemokines like eotaxin.

A limitation of this study is that we performed influenza challenge experiments only with H1N1 NC99 virus. The other two types of influenza viruses that are responsible for morbidity and mortality in the human population are H3N2 and influenza B viruses. We did not test those virus strains in our model at this point, because we did not have mouse-adapted H3N2 and influenza B virus available that matches the strains contained in the TIV we used.

In this study, we combined the influenza mouse model for post-influenza bacterial superinfection with one for TIV vaccination to show that even without efficient induction of HI titers, TIV vaccination protected against lethal bacterial superinfection. TIV vaccination resulted in disease modulation with skewing of host responses to both influenza virus infection and bacterial superinfection. This highlights that other vaccine correlates of protection against influenza are needed beyond HI titers to effectively assess the protective effect of vaccination. In the mouse model we used infection with H1N1 NC99 already resulted in severe morbidity, which in combination with bacterial superinfection resulted in mortality. Therefore, we could not study the effect of bacterial superinfection on the host immune response to influenza virus infection in unvaccinated convalescent mice. To study this, we would need to repeat the experiments with lower doses of challenge virus. These observations also highlight the importance of disease modulation by influenza vaccination in the absence of virus neutralization and suggest that vaccination is still beneficial to prevent (severe complications during) bacterial superinfection even in the absence of virus neutralization. This understanding is important since avoiding bacterial pneumonia by influenza vaccination reduces antibiotics prescriptions [37], which is why influenza vaccination needs to be considered as a public health measure for the battle against antimicrobial resistance [38]. The findings of this study support the encouragement of influenza vaccination for target groups that are at higher risk for complications during influenza infection, like the elderly, who are at risk for developing influenza-associated complications such as post-influenza bacterial superinfections.
